# Real-Life Efficacy of Single-Inhaler Triple Therapy with Budesonide/Glycopyrronium/Formoterol Fumarate in Persistent COPD Users: A Retrospective Database Study

**DOI:** 10.3390/biomedicines13112681

**Published:** 2025-10-31

**Authors:** Bruno Sposato, Leonardo Gianluca Lacerenza, Sara Croce, Elisa Petrucci, Valentina Fabbrini, Laura Giannini, Pasquale Baratta, Alberto Cresti, Alberto Ricci, Claudio Micheletto, Antonio Perrella, Valerio Alonzi, Andrea Serafini, Marco Scalese

**Affiliations:** 1 Pneumology Department, Local Health Unit ‘Toscana Sud-Est’, ‘Misericordia’ Hospital, 58100 Grosseto, Italy; 2 Department of Pharmaceutical Medicine, Local Health Unit ‘Toscana Sud-Est’, ‘Misericordia’ Hospital, 58100 Grosseto, Italy; 3 Corporate Clinical Trials Task Force, Clinical Research Office, Local Health Unit ‘Toscana Sud-Est’, San Donato Hospital, 52100 Arezzo, Italy; 4 Cardiology Department, Local Health Unit ‘Toscana Sud-Est’, ‘Misericordia’ Hospital, 58100 Grosseto, Italyalberto.cresti@uslsudest.toscana.it (A.C.); 5 Division of Pneumology, Department of Clinical and Molecular Medicine, Sant’Andrea Hospital, Sapienza University, 00189 Rome, Italy; 6 Pulmonary Unit, Integrated University Hospital of Verona, 37129 Verona, Italy; 7 Medical Management Department, Local Health Unit ‘Toscana Sud-Est’, ‘Misericordia’ Hospital, 58100 Grosseto, Italy; 8 Institute of Clinical Physiology, Italian National Research Council, 56124 Pisa, Italy

**Keywords:** COPD, budesonide/glycopyrronium/formoterol, exacerbations triple therapy, SABA, oral corticosteroids, antibiotics, hospitalization, long-term, real-life

## Abstract

**Background/Objective**: Single-inhaler triple therapy (SITT) with budesonide/formoterol/glycopyrronium (B/F/G) is an option for COPD patients with frequent exacerbations. We evaluated its long-term efficacy in real life on emergency room visits/hospitalizations (primary endpoints), lung function, oral corticosteroid (OC), antibiotics and salbutamol (SABA) prescriptions (secondary endpoints). **Methods:** The aim of this single-center, retrospective observational study was to evaluate, in 65 COPD patients with recurrent exacerbations, the effects of B/F/G treatment after 18–24 months compared to therapies with LABA/LAMA, ICS/LABA, ICS/LABA + LAMA or other SITT taken in the previous 18–24 months. **Results:** After 22.8 ± 4.6 months, 20.12 ± 4.24 B/F/G packages were prescribed, while packs of other therapies given in the 23.35 ± 4.7 months (*p* = 0.587) before using B/F/G were 15.58 ± 9.8 (*p* = 0.0009). Emergency room visits (0.34 ± 0.56) and hospitalizations (0.52 ± 0.81) during about 2 years of B/F/G therapy were lower compared to the ones during pre-B/F/G treatments (0.65 ± 1.2, *p* = 0.015 and 0.83 ± 1.25, *p* = 0.019, respectively). After B/F/G treatment, the mean FEV_1_% value (48.5 ± 16.7%) was higher compared to that measured after the therapies taken before switching to B/F/G (45 ± 15.3%; *p* = 0.013). Conversely, there were no differences in FVC% values. OCs (2.96 ± 2.6) and SABA (1.41 ± 2.06) packages prescribed during B/F/G were lower than those observed during pre-B/F/G treatments (3.86 ± 2.35, *p* = 0.026 and 2.48 ± 4.57, *p* = 0.046, respectively). No differences in antibiotic prescriptions were observed during both therapies. **Conclusions:** Our real-life evaluation highlighted that B/F/G treatment may be effective, even in the long term, in reducing exacerbations, OC and SABA consumption and in improving lung function in COPD patients with high persistence/adherence to B/F/G compared to other non-persistent inhaled therapies previously taken. Optimizing treatment adherence should be one important goal of COPD patients’ management to maximize the therapy benefits.

## 1. Introduction

Chronic obstructive pulmonary disease (COPD) is a lung progressive inflammatory pathology with heterogeneous presentation that includes emphysema and chronic bronchitis [1]. It is characterized by airflow limitation, dyspnea, frequent coughing or wheezing, excess sputum production and loss of lung function [1]. The main objectives of COPD management are to reduce symptoms and the risk of exacerbations (i.e., acute deterioration in respiratory problems), which are associated with a lower quality of life and poor clinical outcomes, including an increased mortality risk [1]. Inhaled corticosteroids (ICSs) and bronchodilators, including long-acting muscarinic antagonists (LAMAs) or long-acting β2-agonists (LABAs), often used in combination [1], are the standard treatments to reduce airway inflammation and delay symptom progression and loss of lung function, measured as forced expiratory volume in 1 s (FEV_1_). Particularly, in people with COPD on ICS/LABA or LABA/LAMA therapies, the Global Initiative for Chronic Obstructive Lung Disease (GOLD guidelines) recommend stepping up to ICS/LAMA/LABA to patients with frequent exacerbations and/or with persistent major symptoms [1]. Furthermore, GOLD guidelines recognize that single-inhaler therapy (e.g., fixed-dose inhalers) may be more convenient and effective than multiple inhalers. Budesonide/formoterol/ glycopyrrolate (B/F/G) is a fixed triple dose combination therapy available in Italy from January 2022 onward and approved for maintenance treatment in adult patients with moderate-to-severe COPD, who are not adequately controlled with ICS/LABA or LABA/LAMA. Furthermore, it would be preferable to replace MITT with SITT for greater convenience and effectiveness [1].

Several trials demonstrated that it reduced the rate of moderate/ severe exacerbations and improved FEV_1_ compared with GLY/FORM or BUD/FORM dual therapy [2,3]. Furthermore, B/F/G was also able to improve symptoms and HRQoL compared to dual therapies over 24 and 52 weeks in patients with moderate to very severe COPD [4]. However, to date, there is limited evidence in the literature on clinical/functional efficacy of B/F/G in comparison with ICS/LABA, LABA/LAMA or other multiple/single-inhaler triple therapies in the real world. A recent study observed that B/F/G reduced the use of oral corticosteroids and antibiotics during the COPD period of exacerbations and consequently hospitalizations in comparison with dual inhaler therapies [5]. Other recent network meta-analysis observed that B/F/G, by means of MDI, has comparable efficacy to other ICS/LAMA/LABA fixed-dose and open triple combination therapies in reducing exacerbations and improving lung function and symptoms in patients with moderate to very severe COPD [6,7]. Another research highlighted that real-life therapeutic success with B/F/G (namely, no major cardiac or respiratory events) was achieved by 96.5% of patients during a 90-day treatment and by 91.8% during a 180-day therapy, which are significantly higher percentages than other previous inhaler therapies [8].

Therefore, given the limited evidence, we wanted to compare the real-world clinical–functional efficacy of B/F/G to other therapeutic regimens previously used in a population of COPD patients.

## 2. Materials and Methods

### 2.1. Study Design

In the period between 1 January 2021 and 31 December 2024, we searched our pharmaceutical database of USL SUDEST-Tuscany for COPD patients taking B/F/G. Only those who had taken B/F/G for 18–24 months with a persistence/adherence of at least 8 boxes/year were considered. Considering patients who are poorly adherent/persistent to treatment could lead to underestimation of the therapy’s efficacy due to poor adherence to treatment. We then searched for the inhalation therapy, and its number of prescriptions/year, that each patient had been given during the 18–24 months before using B/F/G (ICS/LABA, LABA/LAMA, ICS/LABA+ LAMA or other SITT’s). In the two periods, with the two different treatments, we evaluated the number of emergency room visits and hospital admissions for COPD exacerbations that were primary endpoints. Instead, FEV_1_% and FVC% values measured at the end of each treatment and the number of oral corticosteroids (OCs), antibiotics and Salbutamol (SABA) prescriptions were taken into account as secondary endpoints. The various results obtained with the two therapeutic regimens in the two periods considered (B/F/G and Pre-B/F/G) were compared.

### 2.2. Setting and Participants

As already said, we retrospectively extrapolated from the database mentioned above all patients on SITT who obtained at least 12 boxes of B/F/G (8 packages/year) during the 18–24 months of follow-up. A correct COPD diagnosis had to be made according to GOLD guidelines [1]. In fact, in Italy, the prescription of a triple must be made by means of a written therapeutic plan that requires a precise COPD diagnosis performed with a spirometry before and after the bronchodilator, in addition to the clinical criteria required by the diagnosis. Time of treatment was calculated for each patient from the B/F/G start date until the completion of the eighteenth/twenty-fourth month of therapy. We considered each patient’s therapy with ICS/LABA, LABA/LAMA, ICS/LABA + LAMA or other triples, taken in the 18–24 months before, still using the pharmacy database, together with the prescriptions of OC, antibiotics and SABA for each individual during the two periods with the different treatments. We also collected all the drugs that each patient had taken for other diseases in order to reconstruct the comorbidities associated with COPD. The emergency room visits and hospital admissions for each period and for each subject were searched for in the administrative databases. Spirometric values, obtained at the end of each treatment, were found in the archives of the various spirometers where the patients had undergone the test. The study was approved by “Area Vasta Sudest Ethical Committee (C.E.A.S.V.E.), Azienda Ospedaliera Universitaria Senese and Azienda USL Toscana Sud-Est” (Protocol TRIX, n 26511; 16 September 2024), on the basis that it complied with the declaration of Helsinki and that the protocol followed existing good clinical practice guidelines. Informed consent was obtained from the patients.

### 2.3. Variables and Measurements

Age, gender, comorbidities associated with COPD, number of B/F/G, ICS/LABA, LABA/LAMA, ICS/LABA + LAMA or other SITT’s, oral corticosteroids and antibiotic packages during the two periods with the two different treatment regimens were considered for each patient. The oral corticosteroids taken into account by this study, which can be used in COPD exacerbations, were Betamethasone, Dexamethasone, Methylprednisolone, Prednisolone, Prednisone and Deflazacort. Antibiotics, like penicillins with extended action spectrum, combinations of penicillins with beta-lactamase inhibitors, cephalosporins, macrolides, fluoroquinolones, combinations of sulphonamides and trimethoprim, were considered for this research as they can also be used in COPD exacerbations. Also, prescription numbers of SABA, observed during the two periods of different treatments, were considered as the outcome. B/F/G ≥ 8 packages dispensing was the cut-off that identified a proper persistence/adherence to treatment. Individuals taking less than 8 ICS/LABA/LAMA packs per year were excluded from the study because they were considered poorly adherent to B/F/G. Furthermore, those that had changed their therapy by switching to other inhaler treatments during the period considered were also excluded.

Prescriptions of other drugs indicated for other diseases were also considered for each patient in order to trace the comorbidities that the individuals examined in this study might have been affected by. They were identified by searching for medications taken for various non-COPDs by each patient during B/F/G treatment. Taking at least 3 boxes per year of medications for non-COPD diseases was used to identify the various comorbidities. The following Anatomical Therapeutic Chemical Classification (ATC) codes of drugs used to classify them were as follows: A07, A10, A11/A12/H05/M05, B01/C01/C03C/C03D/C03E/C08D, B03, B03X, C02/C03A/C03B/C07/C08/C09, C10, G04, H03, L01/L02/L03/N02, L04/M01/M04AC01/P01, M04, N03/N04, N05/N06/N07, S01. Such codes identified the following: gastroesophageal reflux/dyspepsia, intestinal disorders, diabetes, osteoporosis, cardiovascular disease, i.e., heart failure, cardiac arrythmias, coronary artery disease, cerebrovascular diseases (considered all together), anemia, renal failure, hypertension, dyslipidemia, prostatic hypertrophy, thyroid disorders, oncology pathologies, autoimmune disorders, hyperuricemia, neurological disorders, psychiatric disorders and glaucoma.

ICD-9 codes of 490, 491, 492, 494 and 496 reported in hospital discharge forms, or 518.81–518.84, 786.0, 786.2 and 786.4 associated with at least one of the secondary diagnoses with the codes 490–491–492–494–496, were regarded as hospitalizations for COPD exacerbations. Visits to emergency rooms were considered when the ICD-9 codes 490, 491, 492, 494, 496, 518.81–518.84, 786.0, 786.2, 786.4, 465.9, 466.0, 466.19, 487.1, 487.8, 493.90 and 493.91 were reported in hospital discharge forms.

Pulmonary function parameters considered were FEV_1_% and FVC%. Other lung function measurements were not available for all patients. Blood eosinophil counts measured during the B/F/G treatment period were also considered for each patient.loem1501

### 2.4. Statistical Analysis

All variables obtained in the two periods with the two different treatment regimens were compared using a paired t test. Data were presented as averages and standard deviations. The primary endpoints were emergency visits and hospitalizations; type I error was adjusted using Holm’s method (or the Holm–Bonferroni method) which is a sequential procedure for multiple hypothesis testing that controls the family-wise error rate (FWER). It involves ordering *p* values from smallest to largest and comparing each one to a progressively adjusted significance threshold. The secondary endpoints (FEV_1_, FVC, oral corticosteroids, antibiotics and SABA prescriptions) were not adjusted for multiple comparisons. *p* values < 0.025 were considered statistically significant for primary endpoints, while those <0.05 were considered statistically significant for secondary endpoints. Comparisons of the number of patients that required at least 2 emergency room visits or 2 hospitalizations and those who had at least 2 prescriptions for OC and SABA or 4 prescriptions for antibiotics in the two different groups were performed by using the chi-square test. The statistical package SPSS 16.0 (SPSS Inc., Chicago, IL, USA) was used for the analysis.

## 3. Results

**Figure 1:**
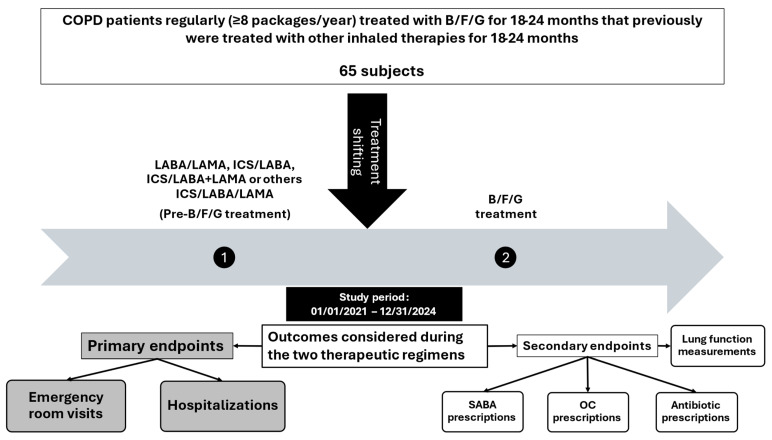
Study protocol.

**Figure 2:**
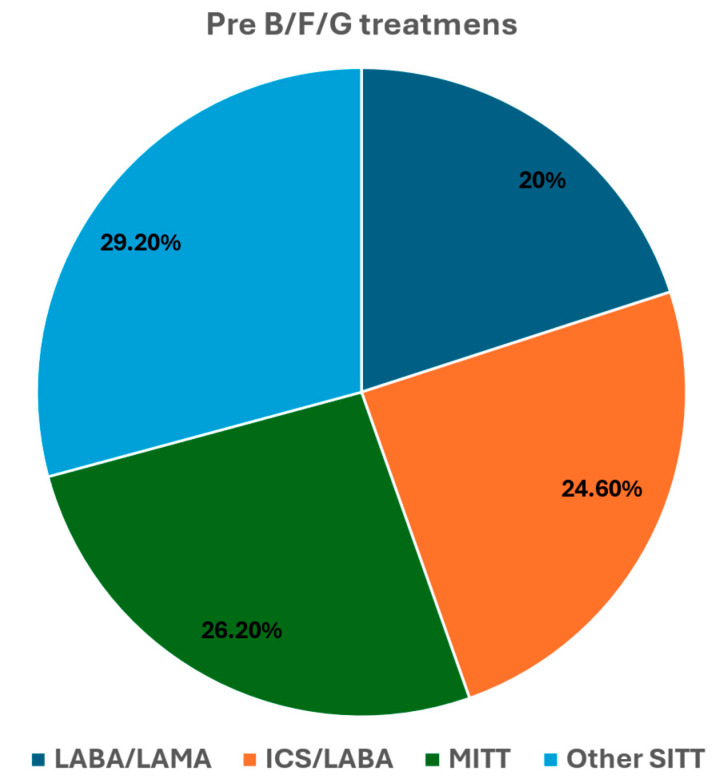
Therapies used in the 18–24 months before B/F/G.

A total of 65 patients were analyzed in this study and their characteristics, like age, sex and comorbidities, are listed in Table 1. The study protocol was described in Figure 1. The therapies that they had taken before treatment with B/F/G are shown in Figure 2. There were LABA/LAMA, ICS/LABA, ICS/LABA + LAMA and an SITT in 20, 24.6, 26.2 and 29.2% of the cases, respectively.

**Figure 3:**
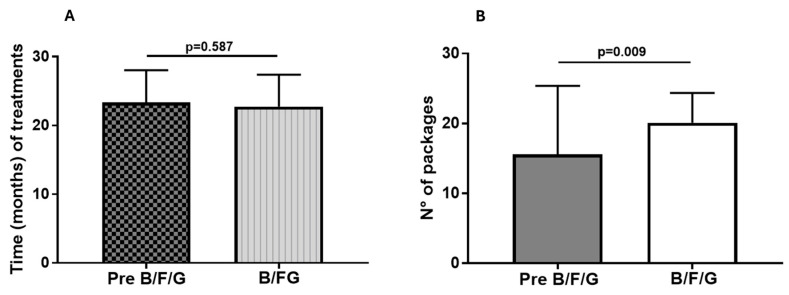
(**A**) Time (mean ± SD) of pre-B/FG treatment and the following therapy with B/F/G; (**B**) number of packages (mean ± SD) of pre-B/F/G treatment (LABA/LAMA, ICS/LABA, ICS/LABA + LAMA, ICS/LABA/LAMA) and the following therapy with B/F/G (i.e., persistence/adherence with the two treatment regimens) during the two different periods.

Treatment times with B/F/G (22.8 ± 4.6 months) and with the previous therapy (23.35 ± 4.7 months) were similar (*p* = 0.587; Figure 3A). Treatment persistence (stay-on-therapy) differed with the two treatment regimens in the two study periods. The number of prescribed boxes of B/F/G during the 23 months of therapy was 20.12 ± 4.24, while the number of boxes of other treatments undergone in the 23 months preceding the use of B/F/G was 15.58 ± 9.8 (*p* = 0.0009; Figure 3B).

**Figure 4:**
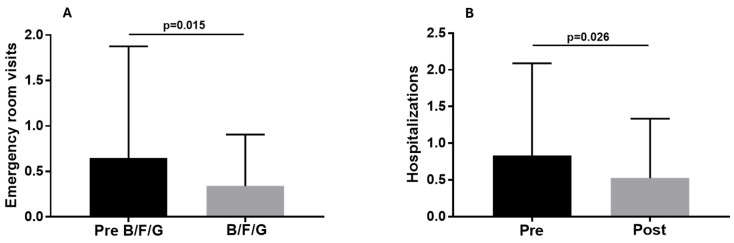
Emergency room visits (**A**) and hospitalizations (**B**) observed during the pre-B/F/G treatment (23.35 ± 4.7 months) and the following therapy with B/F/G (22.8 ± 4.6 months).

**Figure 5:**
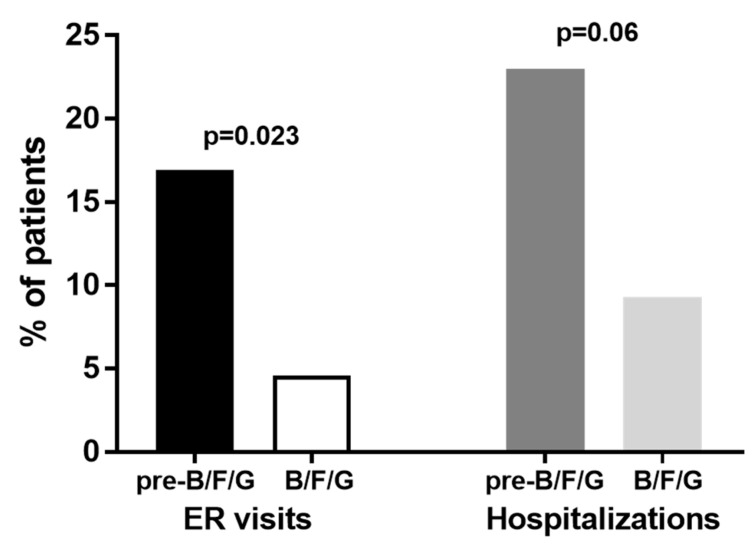
Percentage of patients who had at least 2 emergency room (ER) visits or 2 hospitalizations during the pre-B/F/G treatment (23.35 ± 4.7 months) and the following therapy with B/F/G (22.8 ± 4.6 months).

The number of emergency room visits (0.34 ± 0.56) and hospital admissions (0.52 ± 0.81) during the approximately 2 years of B/F/G were lower than those recorded by patients in the 18–24 months pre-B/F/G (0.65 ± 1.2, *p* = 0.015 and 0.83 ± 1.25, *p* = 0.019, respectively; Figure 4A,B). The number of patients who had at least two emergency room (ER) visits or two hospitalizations during the pre-B/F/G treatment (23.35 ± 4.7 months) was 11 (16.9%) and 15 (23%), while during the following therapy with B/F/G (22.8 ± 4.6 months) the number was 3 (4.6%) and 6 (9.3%), respectively (*p* = 0.023 and *p* = 0.06; Figure 5).

**Figure 6:**
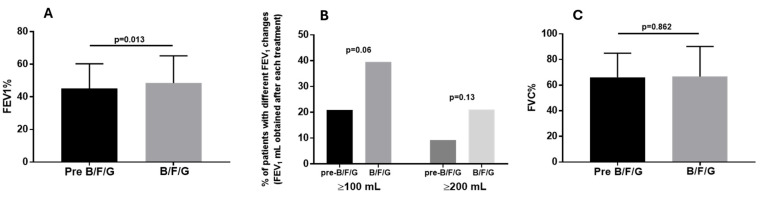
Lung function ((**A**) FEV_1_%; (**C**) FVC%) observed at the end of pre-B/F/G treatment and the following therapy with B/F/G; (**B**) percentage of patients with FEV_1_ change ≥100/mL and ≥200 mL achieved at the end of each treatment (pre-B/F/G and B/F/G, 23.35 ± 4.7 and 22.8 ± 4.6 months, respectively). Lung function was assessed only in 43 patients whose spirometry data were available in the periods considered.

At the end of B/F/G therapy, the mean FEV_1_% value (48.5 ± 16.7%) was higher compared to the one measured after approximately 23 months with the other regimens used before switching to B/F/G (45 ± 15.3%; *p* = 0.013; Figure 6A). On the contrary, there were no differences between the FVC% values obtained at the end of each treatment (B/F/G: 66.7 ± 23.5%; pre-B/F/G: 66.2 ± 18.7%; *p* = 0.862; Figure 6C). The number of patients with FEV_1_ change ≥100/mL and ≥200 mL achieved at the end of pre-B/F/G was 9 (20.9%) and 4 (9.3%) whereas after B/F/G, the number was 17 (39.5%) and 9 (21.1%), respectively (*p* = 0.06 and *p* = 0.13; Figure 6B). Lung function was assessed only in 43 patients whose spirometry data were available in the periods considered.

**Figure 7:**
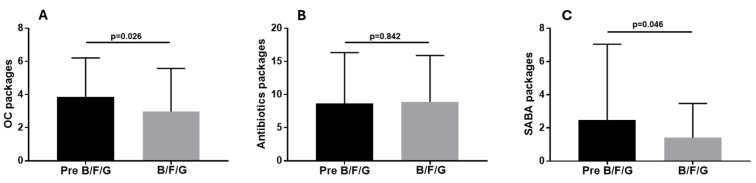
OC (**A**), antibiotics (**B**) and SABA (**C**) packages prescribed during the pre-B/F/G treatment (23.35 ± 4.7 months) and the following therapy with B/F/G (22.8 ± 4.6 months).

**Figure 8:**
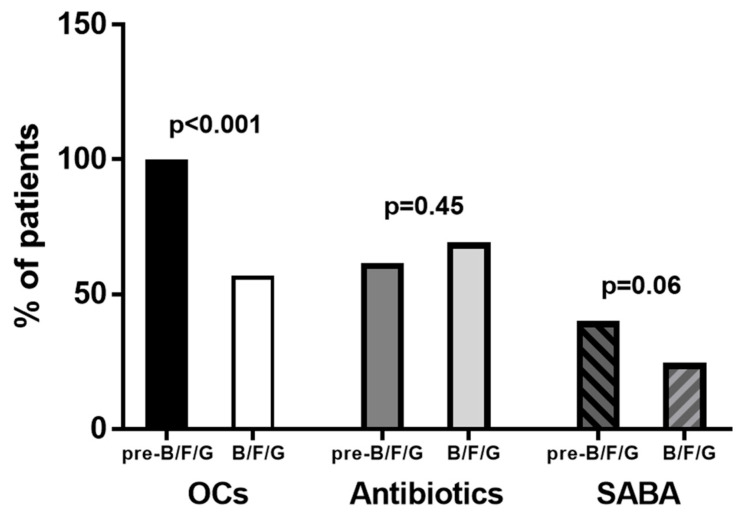
Percentage of patients who had at least 2 prescriptions for OC and SABA or at least 4 prescriptions for antibiotics during the pre-B/F/G treatment and the following therapy with B/F/G (23.35 ± 4.7 and 22.8 ± 4.6 months, respectively).

The number of OC (2.96 ± 2.6) and SABA (1.41 ± 2.06) prescriptions during the 18–24 months of B/F/G therapy was lower than the one observed with the therapeutic approaches performed during the 18–24 months preceding the use of B/F/G (3.86 ± 2.35, *p* = 0.026 and 2.48 ± 4.57, *p* = 0.046, respectively; Figure 7A,C). Antibiotic prescriptions remained stable with B/F/G and with the other therapies previously used (*p* = 0.842; Figure 7B). The number of patients who had at least two prescriptions for OC and SABA during the pre-B/F/G (23.35 ± 4.7 months) was 65 (100%) and 26 (40%) whereas during the following therapy with B/F/G (22.8 ± 4.6 months), the number was 37 (56.9%) and 16 (24.6%) (*p* < 0.001 and *p* = 0.06, respectively; Figure 8). The percentage of subjects who had at least four prescriptions for antibiotics was similar in the two groups (*p* = 0.45; Figure 8).

## 4. Discussion

According to our study, switching to single-inhaler B/F/G therapy from ICS/LABA, LAMA/LABA, ICS/LABA + LAMA or other SITT’s was more effective in decreasing emergency room visits and hospitalizations for COPD exacerbations as well as in improving lung function and especially in reducing the use of oral corticosteroids (mainly used for exacerbations) and SABA prescriptions. It should be emphasized that these results were observed after a long follow-up of 18–24 months of treatment. There are no such long-term studies in the literature dealing with B/F/G; other analyses of this triple therapy stop at a maximum of 12 months. The benefits observed up to 24 months suggest a persistence of efficacy of this triple therapy even in the long term. Furthermore, similarly to an observational retrospective analysis of an Italian administrative healthcare database [9], we found that COPD patients receiving B/F/G were older, had more comorbidities, particularly CVD/hypertension and gastroesophageal diseases, and a very impaired lung function. This is to underline that the improvement was obtained in patients with severely compromised lung function and with multi-comorbidities, which suggests that early treatment with B/F/G in less severe patients may lead to better results over time.

Dual therapy with LABA/LAMA or ICS/LABA was the most common maintenance treatment prior to escalation to BGF (44.6%), while the other patients (55.4%) switched from other triple therapies (MITT or SITT). The results were similar to those obtained by other authors [10]. This may indicate a lack of effectiveness of these previous treatment options or a preference for a single triple B/F/G inhaler by patients, which is supported by the fact that such subjects were more persistent to treatment (a possible greater adherence) with this inhaler.

We observed that B/F/G was particularly effective in reducing especially severe exacerbations measured as emergency department visits, hospital admissions and OC use. Indeed, the number of emergency room visits/hospitalizations and the number of OC boxes, as well as the number of subjects who underwent at least two ER visits/hospitalizations or who were prescribed at least two OC packages, were significantly lower with B/F/G. Reducing the risk of COPD exacerbations is the primary goal of therapy in this disease [1]. Exacerbations, particularly severe ones, are known to be a risk factor for COPD mortality [11,12]. It has been ascertained that there is an estimated 11-fold increased risk of death following a severe exacerbation compared with no flare-ups, and an approximately 2-fold increased risk of death following one moderate exacerbation compared with no flare-ups [13]. In fact, by significantly reducing exacerbations, B/F/G, after one year of treatment, compared to the use of LABA/LAMA, can also reduce all-cause mortality as demonstrated by the ETHOS study [14]. The benefit in terms of exacerbation reduction with B/F/G may be caused by the use of budesonide with possible repercussions on mortality, especially those due to cardiovascular causes, as several studies seem to highlight [14,15,16,17].

An original aspect of this study was that it showed that B/F/G significantly reduces SABA consumption compared to previous therapies. We know that SABA overuse is associated with the perception of greater dyspnoea [18]. This would also confirm the efficacy of B/F/G on symptoms in accordance with what has already been observed by other authors who have found a significant improvement in mMRC and CAT in responders to B/F/G, compared to what was obtained with non-SITT pre-treatment [19].

After almost 2 years of therapy with B/F/G, we also observed a slight improvement in lung function (FEV_1_) compared to pre-treatment. The percentage of subjects showing an improvement of at least 100 mL (a significant increase in subjects with severe COPD) was higher in subjects treated with B/F/G. The ETHOS study also observed B/F/G benefits on lung function maintaining this effect even up to 52 weeks of treatment [20]. Certainly, after a period of at least 2 years, we have not observed any deteriorations in lung function, leading us to hypothesize a possible slowing down or even a stoppage of the disease progression (lung function decline), which is a COPD characteristic [1].

One of the reasons for the greater effectiveness of B/F/G could be due to the greater persistence/adherence to this treatment rather than to other therapeutic regimens previously taken. Our study enrolled only patients with high compliance to treatment (at least eight prescriptions/year) excluding subjects not adhering to B/F/G, with the aim of selecting subjects who were undergoing this therapy regularly. The results obtained were compared with those found in the previous 18–24 months with a different treatment that was taken with a lower persistence. The greater treatment persistence with B/F/G, compared to the previous therapy, suggests that these patients were more motivated to take and continue with B/F/G, a motivation probably supported by its greater efficacy compared to other previous treatments. In fact, approximately 20 B/F/G packs were prescribed in 18–24 months, while an average of approximately 15 boxes of the other therapies were given in the 18–24 months before B/F/G. This leads us to hypothesize that adherence to treatment, which was better with B/F/G, according to our study, in comparison to pre-B/F/G treatment, may play a fundamental role in the therapy of COPD with SITT. As already said, the benefits of B/F/G treatment, at least in part, may be due to a higher persistence/adherence. Several studies showed that SITT can lead to a more significant persistence/adherence medication compared with an MITT regimen [21,22,23,24,25,26] and, according to our study, also compared with ICS/LABA, LABA/LAMA and other SITT’s. Such high compliance is crucial for achieving optimal clinical outcomes [27,28,29,30]. Decreased adherence in COPD is associated with worse outcomes, with increased risk for exacerbations and long-term mortality [27]. SITT treatment gives an opportunity to improve poor therapy persistence/adherence by removing barriers such as complexity, dosing frequency, number and variety of medications and ease of inhaler use [31]. Therefore, switching to a B/F/G therapy in subjects who are poorly adherent to other inhaled treatments could be a choice to increase compliance with repercussions of greater clinical/functional efficacy.

A synergistic effect due to the simultaneous administration of drugs may be one of the mechanisms underlying the higher effectiveness of a single-inhaler therapy compared to the one obtained via multiple devices. We know that bronchodilator combinations (LAMAs and LABAs) have also been demonstrated to exhibit a superior efficacy due to their combined action mode when compared to monotherapy [32,33,34]. This has also been demonstrated with the triple beclomethasone/formoterol/glycopyrronium combination that induced an increased bronchorelaxant effect in medium and small human airways [35]. Consequently, the simultaneous intake of inhaler medications should always be considered in COPD treatment in order to obtain a greater benefit.

A limitation of this study is the number of subjects recruited. However, we believe that, despite the limited number of patients, the results obtained from our study are generalizable to the highly adherent patients of the COPD population. The greater adherence observed in patients switching to B/F/G is certainly the most important factor influencing the clinical efficacy of this treatment. Achieving greater adherence should be the primary goal of any therapy in order to attain to a greater clinical efficacy.

## 5. Conclusions

In conclusion, our real-life study highlighted that persistence/adherence to B/F/G therapy appears to be more effective than irregular non-B/F/G treatment in reducing COPD exacerbations in terms of emergency room visits/hospitalizations and OCs consumption and in improving lung function and symptoms seen with the reduction in SABA consumption. Such benefits can be observed even after 18–24 months. A greater adherence to B/F/G may be the main reason of its efficacy. Optimizing medication adherence should be one of the goals of the management of COPD patients to maximize the benefits of therapy that could be especially achieved with a triple therapy with a single inhaler.

## Figures and Tables

**Table 1:** Characteristics of patients.

Age	72.5 ± 9.04
Sex (M/F)	38/27
Ex smokers	63 (96.9%)
Smokers	2 (3.1%)
GOLD stage II	15 (23.1%)
GOLD stage III	31 (52.3%)
GOLD stage IV	16 (24.6%)
Blood eosinophils count ≥ 300/µL (evaluated on 58 patients)	7 (12%)
Blood eosinophils count between 200 and 299/µL (evaluated on 58 patients)	13 (22.4%)
Blood eosinophils count between 100 and 199/µL (evaluated on 58 patients)	38 (65.6%)
Cardiovascular diseases	52 (80%)
GER/DY	52 (80%)
Hypertension	51 (78.4%)
Dyslipidemia	24 (36.9%)
Cancer	22 (33.8%)
Psychiatric disorders	20 (30.7%)
Hyperuricemia	18 (27.7%)
Diabetes	15 (23.1%)
Prostatic hyperplasia	14 (21.5%)
Anemia	12 (18.4%)
Others	32 (49.2%)

## Data Availability

The data are not available due to ethical and regulatory restrictions.
